# Adherence to Trained Standards After a Faculty Development Workshop on “Teaching With Simulated Patients”

**DOI:** 10.3205/zma001122

**Published:** 2017-10-16

**Authors:** Julia Freytag, Henrike Hölzer, Ulrike Sonntag

**Affiliations:** 1 Charité - Universitätsmedizin Berlin, Prodekanat für Studium und Lehre, Berlin, Germany; 2 Medizinische Hochschule Brandenburg, Neuruppin, Germany

**Keywords:** Faculty development, Evaluation, Observational study, Checklist, Teaching Standards, Teaching with Simulated Patients

## Abstract

**Background: **Nowadays, faculty development programs to improve teaching quality are considered to be very important by medical educators from all over the world. However, the assessment of the impact of such programs rarely exceeds tests of participants’ knowledge gain or self-assessments of their teaching behavior. It remains unclear what exactly is expected of the attending faculty and how the transfer to practice may be measured more comprehensively and accurately.

**Method:** This study evaluates how specific teaching standards were applied after a workshop (10 teaching units) focusing on teaching communication skills with simulated patients. Trained observers used a validated checklist to observe 60 teaching sessions (held by 60 different teachers) of a communication skills course integrating simulated patients. Additionally, we assessed the amount of time that had passed since their participation in the workshop and asked them to rate the importance of communication and social skills in medical education.

**Results: **The observations showed that more than two thirds of teaching standards were met by at least 75% of teachers. Fulfillment of standards was significantly connected to teachers’ rating of the importance of communication and social skills (*t_b_*=-.21, *p*=.03). In addition, the results suggest a slight decrease in the amount of fulfilled standards over time (*r*=-.14, *p*=.15).

**Conclusions: **Teachers' adherence to basic teaching standards was already satisfying after a one-day workshop. More complex issues need to be re-addressed in further faculty development courses with a special focus on teachers’ attitude towards teaching. In future, continuing evaluations of the transfer of knowledge and skills from faculty development courses into practice, preferably including pre-tests or control groups, are needed.

## Zusammenfassung

**Hintergrund: **Weltweit werden hochschuldidaktische Fortbildungen zur Verbesserung der Qualität der Lehre von Lehrenden als sehr wichtig angesehen. Zumeist werden die Effekte dieser Programme jedoch lediglich über den Wissenszuwachses der Teilnehmenden oder eine Selbsteinschätzung ihres (veränderten) Lehrverhaltens erhoben. Offen bleibt, was genau von den Teilnehmenden erwartet wird und wie der Transfer des Gelernten in die Praxis umfassender und genauer gemessen werden kann.

**Methoden:** In dieser Studie wird evaluiert, ob spezifische Qualitätsstandards nach der Teilnahme an einem Workshop (10 UE) zur Vermittlung kommunikativer Kompetenzen mit Simulationspatient*innen (SP) in der Praxis eingehalten werden. Geschulte Beobachterinnen nutzten eine validierte Checkliste um in 60 Kursen zu kommunikativen und sozialen Kompetenzen, in denen SP eingesetzt wurden, zu hospitieren (bei 60 verschiedenen Lehrenden). Zusätzlich wurden der Zeitraum, der seit der Workshopteilnahme jeweils vergangen war sowie die Einstellung der Lehrenden zur Wichtigkeit kommunikativer und sozialer Kompetenzen in der medizinischen Ausbildung erfasst.

**Ergebnisse: **In den Hospitationen zeigte sich, dass mehr als zwei Drittel der Qualitätsstandards von mindestens 75% der Lehrenden eingehalten werden. Die Anzahl an erfüllten Standards korrelierte signifikant mit der Bewertung der Wichtigkeit kommunikativer und sozialer Kompetenzen durch die Lehrenden (*t_b_*=-.21, *p*=.03). Zusätzlich deuten die Ergebnisse eine Abnahme der Anzahl eingehaltener Standards über die Zeit an (*r*=-.14, *p*=.15).

**Schlussfolgerungen:** Bereits nach Teilnahme an einem nur eintägigen Workshop war die Einhaltung grundlegender Qualitätsstandards auf zufriedenstellendem Niveau. Komplexere Standards müssten in weiteren hochschuldidaktischen Kursen vertieft werden, dabei sollte ein Fokus auf der Einstellung der Lehrenden zur Relevanz kommunikativer Kompetenzen liegen. Zukünftig sind fortlaufende Überprüfungen des Transfers von Wissen und Fertigkeiten aus didaktischen Kursen in die Lehrpraxis nötig – bevorzugter Weise unter der Einbindung von Prä-Tests oder Kontrollgruppen.

## 1. Introduction

These days, faculty development programs focusing on all different aspects of teaching are common at medical schools around the globe and are considered a valuable help by many who teach in medical education. Descriptions of such qualifications – whether they are one-time interventions or seminar series – often include evaluations, which report participants’ satisfaction, their knowledge gain and/or self-reports of changes in teaching behavior. Evaluations using different methods, like experts observing the teaching behavior, are used less often [1], [2], [3]. This means that little is known about how teachers implement what they learned in faculty development workshops into their teaching. Especially looking at teaching with simulated patients, as a very specific teaching method, there are only few publications that describe and evaluate workshops or seminars designed to introduce faculty to such methods [4]. When evaluating such a workshop at our own faculty, we wanted to focus on actual teaching behavior and the adherence to specific teaching standards.

A workshop of 10 teaching units (à 45 minutes) called “Teaching with simulated patients” was introduced, after the curriculum at Charité – University Medicine Berlin was modified in 2010 and a communication skills course with 25 different simulated patients (SPs) scenarios was implemented [5]. This change caused an enormous need for qualified teachers of social and communication skills at a time where there were only few available. The completion of the workshop is mandatory for every teacher of a course called KIT. KIT stands for the German translation of “Communication, interaction, teamwork”. It is obligatory, taught in small groups and focuses on the most important social and communication skills needed by physicians, following the recommendations of international consensus statements [6], [7]. A special feature of this course is the integration of simulated patients.

In the workshop “Teaching with simulated patients” we introduce several standards on how to teach with simulated patients [5]. Included is basic knowledge about the methodology of simulated patients, the use of feedback and the basic tasks of an instructor and a facilitator [5], [8]. Furthermore, the participants have the opportunity to practice what they learned in two micro-teachings with SP scenarios used in KIT and receive feedback afterwards. These micro-teachings are considered helpful [9] – not only because new skills can be immediately trained – but also because participants can empathize with the learners’ experience of being observed and assessed [10]. The workshop is held on one full or two half-days, the average number of participants is ten. In an earlier study participants rated the quality of the workshop as very high [3].

Regarding Kirkpatrick’s four levels (reaction, learning, behavior, results [11]), which are often used to classify evaluations of faculty development programs [1], [2], this is an evaluation on level one, concentrating on the participants reaction – their satisfaction – to the workshop. As mentioned above we sought to take this evaluation further and decided to conduct a study to observe the behavior, respectively the behavior change in trained teachers, thus an evaluation on level three. In Steinert et al.’s systematic review 72% of studies report outcomes on level three, but about one third of those exclusively rely on self-reported changes in behavior [1]. But self-assessment is not an easy task, especially if a field of expertise is new to a person [12], [13], [14]. Therefore other measures, like assessments by experienced observers, students or colleagues should be considered as well. However, only few studies integrated such assessments from different sources in recent years to evaluate teaching skills [15], [16]. Consequently, this study – using trained observers and a validated checklist – contributes an important additional insight in this area.

On top of that, we wanted to consider other variables that potentially influence the transfer of learned teaching standards into practice. Based on the literature we identified three variables: time passed since participation in the workshop [1], [17], attitude towards the importance of teaching communication skills to medical students [18], [19] and teachers’ self-perceived need for further training [20]. The amount of *time since participating* in a workshop is used in some studies to investigate if faculty development has sustainable effects [17]. This question is often linked to the duration of the program [1], [2]. With the evaluation of our one-day course we wanted to gain further insight into the effectiveness of such short courses. The question towards teachers’ *self-perceived need for more training* tries to identify their self-efficacy, meaning their self-evaluation, considering their teaching abilities. Literature from different backgrounds, including Bandura’s social cognitive theory, has linked the evaluation of one’s own (teaching) abilities to actual (teaching) behavior [20], [21], [22], [23]. A similar connection between *attitudes* and behavior in different areas was shown in the literature [19], [24]. Furthermore, it is supported by our own experiences working in the field of faculty development. These experiences strongly suggest the importance of teachers’ attitude towards teaching, especially when teaching topics outside the traditional range of subjects or when using innovative methods. This leads to our basic assumption, that – in order for a faculty development workshop to have the desired effect – teachers need to be sensitized to the topics’ importance as well as to the adequacy of the teaching methods. Thus, only dedicated teachers expect a worthwhile outcome from teaching communication skills. Due to that reason, our workshop includes a presentation and a discussion of research results which prove the benefits of communication skills training as well as the implementation of simulated patients. This hypothesis is supported by Bandura’s social cognitive theory as well, as it describes how outcome expectancy plays an important role when it comes to actual performance [23].

To summarize, this study aims to answers the following questions: To what extent do teachers adhere to trained teaching standards for working with SPs? And is adherence to those standards connected to the factors: *time passed since workshop, personal need for further training or importance of communication and social skills in medical education* (rated by teachers)?

## 2. Method

### 2.1. Study Design 

The study is a quantitative study with a quasi-experimental design. Four trained observers collected data during systematic, non-participant observations of KIT sessions in 2013 and 2014. Additionally, teachers were asked to fill out a questionnaire.

#### 2.2. Assessment Procedure 

At first, randomly selected teachers were informed about the study and asked via e-mail for their and their students’ permission to be observed during one of their teaching sessions. If they agreed to be observed, a date was scheduled. Observers were blinded regarding the point in time when teachers had participated in the workshop. Furthermore, observers did not know the participants. They were present for the whole duration of the courses (two to three hours), filled out an observation checklist and handed out questionnaires to the teachers to be filled out at the end of the session. 

#### 2.3. Instruments 

##### 2.3.1. Observation checklist

The checklist was developed at Charité–University Medicine Berlin to evaluate the workshop called “Teaching with simulated patients” [25], because no instrument fitting our purpose could be found when conducting a literature search. The checklist contains 39 items in 7 categories and represents the content, respectively the learning objectives of our workshop and emphasizes our quality standards, for example learner-centered teaching, orientation towards learning objectives and high-quality feedback. The instrument was discussed with the facilitators of the workshops and other faculty development experts to secure its content validity. For some categories, e.g. *Feedback*, a great amount of literature and even validated checklists already exist. In those cases, these were used as a basis for the items of the checklist [8], [26]. Apart from that, we made sure we followed the methodological guidelines for designing an observation checklist, for example that all items need to describe something observable or that items should not overlap each other [27].

The seven item-categories are:

1. Beginning of session (4 items)
2. Interaction with SP (18 items)
3. Feedback (8 items)
4. Content (1 item)
5. Facilitation (4 items)
6. End of session (2 items)
7. Structure of session (2 items)

A pilot study (N=8) was conducted to check the instruments’ feasibility and to identify missing or superfluous items as well as unclear item formulations. Afterwards some items were rephrased for clarification, some items were dichotomized (can only be rated “fulfilled” or “not fulfilled”) and a few new sub-items were established. Some items were fulfilled in all sessions (e.g. “The SP leaves the room when simulation is finished”), nonetheless we decided that they should remain in the checklist, because we see them as crucial parts of a successful SP-interaction. 

The 39 items of the final version (see Attachment 1) can be rated using a scale with four options (“fulfilled”, “partly fulfilled” [if applicable], “not fulfilled”, “not applicable”). Four items are sub-items, which are only applicable in some cases (e.g. if a discussion takes place). The checklist closes with an additional item called “global rating of teaching performance”, which is measured on a five-point Likert scale (1=*very good* to 5=*very poor*).

Observers (two psychologists, two health professions educators) were trained in a two-hour training session where the concept and handling of the checklist were explained. Furthermore, we tested the interrater agreement of our four observers, by letting them rate eight KIT courses in pairs and comparing their ratings (as part of the pilot study). Evaluation using the measure of Percent Agreement [28], [29], showed an initial agreement on 80% of items, which is considered satisfying. 

##### 2.3.2. Questionnaire for teachers

Teachers were asked for demographic information (*gender, age, profession* and* time passed since participation in workshop*) as well as their opinion on the importance of communication and social skills in medical education (five-point Likert scale: 1=very *important* to 5=very *unimportant*). Furthermore, they were asked, if they felt they needed more training in teaching those skills (*yes/no*). This questionnaire was also tested in our pilot study, no further adjustment was needed. 

#### 2.4. Sample

60 teachers participated in the study (out of 170 trained teachers who taught KIT at that time). Of these 60, 37 (62%) were female; their age ranged from under 30 to 59, most participants were 30 to 39. About two thirds of participants were physicians (62%); the remaining participants were psychologists (27%) or belonged to a profession which was not further specified (11%). The amount of time that passed since they took part in the workshop is depicted in Table 1 (Tab. 1). 

#### 2.5. Statistical Analysis

The statistical analysis was executed using SPSS, Version 22 (Armonk, NY: IBM Corp.) For every item we calculated the percentages of teachers that fulfilled, partly fulfilled and did not fulfill it, as well as the mean and the range of the total number of fulfilled standards by using descriptive statistics. To test for group differences, we used t-tests, correlations were calculated with Pearson's r or Kendall’s tau-b (*t_b_*) as measures. The alpha level was set at 0.05. To be able to judge the meaningfulness of the results more accurately, effect sizes were calculated.

## 3. Results

### 3.1. Fulfilment of Standards

All in all, over two thirds of the 35 teaching standards (without counting the 4 sub-items) were either fulfilled or partly fulfilled by over 45 teachers (75%). The mean number of standards that were fulfilled (partly or fully) was 29.1 out of 39 (*SD*=4.5); the total number of standards that teachers adhered to ranged from 17 to 37 standards (including sub-items). We divided the standards into categories, based on how many teachers were able to fulfill them (see Attachment 2). We identified 14 standards out of 35 that were fulfilled nearly all teachers (by at least 76%). 12 standards were fulfilled by most of teachers (51-75%) and 7 only by less than half (26-50%). The category with the lowest adherence-rate consisted of 2 standards, which maximally 25% of teachers implemented into their teaching. Apart from that, calculations showed a significant correlation between the number of fulfilled standards and the global rating of teaching performance by the observers (*t_b_*=-.5, *p*<.001).

#### 3.2. Time Passed since Workshop

The number of semesters since teachers had participated in the workshop did not correlate significantly with the number of standards that teachers fulfilled (*r*=-.14, *p*=.15). 

#### 3.3. Importance of Communication and Social Skills

The majority of teachers rated communication and social skills in medical education as very *important* (70%) or *important* (28%). Only 2% of teachers were *undecided* about the importance of those skills. The number of standards that were fulfilled correlated significantly with teachers’ opinion on how important communication and social skills in medical education are. The correlation with the fulfilled standards was *t_b_*=-.21 (*p*=.03).

#### 3.4. Need for Further Training

Exactly 50% of teachers (30) affirmed a need for further training on how to teach communication skills. There was no significant difference between teachers that did not feel a need for further training and those with need for further training regarding the amount of standards they fulfilled (*t*(54)=.44, *p*=.33, effect size *r*=.06). 

## 4. Discussion

The overall conformity with standards can be considered satisfying; nonetheless the range of standards met between different teachers is rather big. This shows that teachers reached diverse levels of teaching proficiency and that we need to tailor future trainings to different kinds of needs. By taking a closer look at the standards with rather high and rather low adherence, a certain pattern can be found. Deficiencies are mostly found in areas which could be described as *communication of goals and structuring/facilitation of session*. These include items like *teacher introduces learning objectives, teacher activates prior knowledge of students or teacher initiates a final feedback round* at the end of the session. Areas with high adherence are for example* facilitation of interaction with SP* and *giving feedback*. Most of the standards of the latter two areas are also known and reinforced by the SP and the students; therefore, it seems that those standards that must be fulfilled by the teacher all by himself/herself are the standards where we observed lower adherence rates. Some reasons for that might be that teachers have either forgotten these tasks or they avoid these tasks because they find them too difficult or they simply do not see them as important parts of a productive session. Further investigations could shed light on this question and identify potential barriers as well as participants’ commitment to implement the learned standards. As the literature proposes, the concept of *commitment to change* is a valuable approach to investigate the impact of continuing medical education on participants’ daily practice [30], [31].

No statistically significant connection between the time that went by since teachers participated in the workshop and the amount of standards they met could be found. Nonetheless, the correlation of time and achievement of standards had a small to medium effect size, suggesting that the longer the time since the workshop took place, the smaller the number of standards met. Former studies and reviews point in the same direction, with longer programs, like seminar series, showing better and more sustainable results [1]. With workshops as one-time interventions, different authors suggest refresher or follow-ups to maintain the quality of teaching [32], [33].

The importance that teachers assigned to communication and social skills in medical education was correlated significantly with the number of standards teachers adhered to (small to medium effect size) in the direction that teachers who found these skills (very) important met more standards. It is important to note, that teachers' rating of importance of communications skills showed only small variance (98% rated it *important* or very *important*). Nonetheless, even this small difference between *important* and very *important* seems to be connected to teaching behavior. 

The need for further training showed no connection to the amount of fulfilled standards. This shows that – in this case – the teachers’ self-perceived need for further training cannot be directly linked to their actual teaching performance. This is relevant when conducting needs assessment for faculty development initiatives. In addition to the teachers’ subjective need for training, the needs of different other stakeholders, like students and simulated patients [34] should be taken into account and carefully weighed.

### 4.1. Limitations

There are certain limitations to this study. To begin with, we could not conduct a pre-test or use a control group with teachers, who had not yet been trained, because it is not justifiable to let some students be taught by untrained teachers while others are taught by experts. Therefore, it could be debated, whether the adherence to the teaching standards is a direct consequence of our workshop. Nonetheless, most standards are specifically tailored to the local teaching setting with SPs, which increases the likelihood that they were learned in the workshop. The alignment between the learning objectives of this specific workshop and the checklist items helped with measuring the desired teaching behavior. 

Another limitation is that every teacher was observed only once and that the observation itself may have had an effect on the teaching performance [35]. With regard to the observation checklist, one has to keep in mind that the included standards are basic requirements of effective implementation of SPs, but that they alone cannot guarantee a high teaching quality. For example, even if an interaction between student and SP is followed by feedback, reflection and group discussion, this does not necessarily mean that the most relevant issues will be discussed in the most productive way. 

One last limitation refers to the sample and its size. The size (*N*=60) was rather big, considering the fact, that the whole population of trained teachers counted only 170 people at that time. Nevertheless, 60 observations seem to have been insufficient to produce significant results in some cases. In addition, 27 teachers did not give permission to observe their teaching sessions, which threatens the representativeness and explanatory power of the results. Therefore, further research with more participants is required and observation of the KIT sessions should become a regular part of the faculty development process. If refreshment workshops are established, studies need to examine their impact, preferably by using a study design with pre- and post-test.

## 5. Conclusion

In summary, this study shows that in a one-day workshop on teaching with simulated patients a substantial number of learning objectives can be reached and that trained observers are able to register the application of teaching standards afterwards. Nonetheless, certain, more complex teaching skills and tasks need further attention in additional or refreshment workshops. The importance of teaching standards needs to be made more explicit to teachers as well. Furthermore, when training teachers for medical education, their attitude towards teaching, or rather teaching a specific topic like communication skills, should be taken into consideration, because – as our results indicate – teaching quality is connected to the teachers’ attitude towards a topics’ relevancy. To conclude, while well designed single-day faculty development workshops can already produce significant effects on teaching, those effects may be enhanced considerably when crafting a longitudinal faculty development program that also tends to aspects such as teachers’ attitudes and professional development.

## Acknowledgement

The authors wish to thank Judith Moerschner and Sandy Fach for their contribution to this study as trained observers collecting data.

## Funding

The conception and implementation of the described workshop was partly supported by the German Federal Ministry of Education and Research (BMBF; grant number: 01PL11036).

## Ethical approval

The study was exempt from ethical approval by the ethics committee of Charité–University Medicine Berlin (No. EA1/170/14).

## Data

Data for this article are available from the Dryad Digital Repository: http://dx.doi.org/10.5061/dryad.13250 [36]

## Competing interests

The authors declare that they have no competing interests. 

## Supplementary Material

Observation checklist

Table B1: Percentage frequency of observer's ratings of the checklist items (N=60) Table B2: Percentage frequency of observer’s ratings of overall teaching performance (N=60)

## Figures and Tables

**Table 1:**
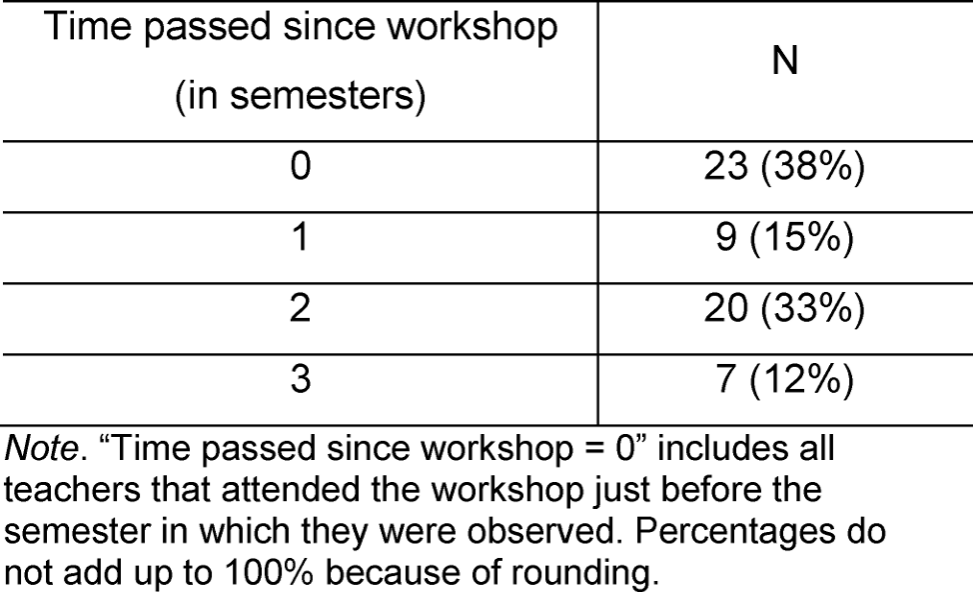
Overview of the time passed since teachers’ participation in workshop (N=60, [one missing value]).
