# Supplementary material for: Adherence to Trained Standards After a Faculty Development Workshop on “Teaching With Simulated Patients”
Source: GMS J Med Educ. 2017 Oct 16;34(4):Doc45. doi: 10.3205/zma001122 (PMC5654119; doi:10.3205/zma001122)
Supplement: Observation checklist [file JME-34-45-s-001.pdf]

Observation Checklist

No.	Category/ Items	*Yes*	*Partly*	*No*	*Not applicable*
1	***Beginning of session***<br>*The teacher…*				
1.1	*… asks about teamwork in PBL course.*				
1.2	*… introduces learning objectives.*				
1.3	*… introduces schedule for session.*				
1.4	*… asks about current issues of group members.*				
2	***Interaction with simulated patient (SP)***<br>*The teacher…*	*Yes*	*Partly*	*No*	*Not applicable*
2.1	*…appoints one student to talk to the SP.*				
2.2	*…makes sure the group knows the important facts about the SP-case.*				
2.3	*… discusses learning objectives of the SP-case in group.*				
2.4	*… activates prior knowledge of students.*				
2.5	*… distributes specific observation tasks.*				
2.6	*A setting for the simulation is prepared.*				
2.7	*… talks to SP prior to simulation.*				
2.8	*… stays in his/her role as an observer during simulation.*				
2.9	*SP leaves the room when simulation is finished.*				
2.9.1	*… makes sure the SP leaves the room when simulation is finished.*				
2.10	*… makes sure nobody gives feedback until SP is back in the room.*				
2.11	*A setting for the feedback is prepared.*				
2.12	*… makes sure feedback is given in the recommended order.*				
2.13	*… makes sure the student summarizes what she/he has learned.*				
2.14	*SP is involved in evaluation and discussion.*				
2.15	*A discussion takes place.*				
2.15.1	*… makes sure that evaluation stays separated from discussion.*				
2.15.2	*... facilitates group discussion & reflection.*				
3	***Feedback culture***<br>*The teacher…*				
3.1	*... starts with a positive comment.*				

No.	Category/ Items	*Yes*	*Partly*	*No*	*Not applicable*
3.2	*… provides „I-messages“*				
3.3	*… reports specific observations without interpreting.*				
3.4	*… combines criticism with constructive suggestions for improvement.*				
3.5	*… only comments on changeable behaviors.*				
3.6	*… limits feedback to a few important comments.*				
3.7	*All the students follow the rules of giving feedback.*				
3.7.1	*… intervenes when students do not follow the rules of giving feedback.*				
4	***Content***<br>*The teacher…*				
4.1	*… orients him-/herself towards the semester's learning objectives.*				
5	***Facilitation***<br>*The teacher…*				
5.1	*… acts as a facilitator.*				
5.2	*… summarizes important aspects.*				
5.3	*… activates students to participate.*				
5.4	*…visualizes important aspects.*				
6	***End of session***<br>*The teacher…*				
6.1	*… initiates a final feedback round.*				
6.2	*… provides an outlook on the next session.*				
7	***Structure of session***<br>*The teacher…*				
7.1	*… initiates a break.*				
7.2	*… adheres to the timeframe.*				

Overall rating of teaching performance (1 = very good – 5= very poor)

1	2	3	4	5

Note: For all checklist items, complementary behavioral anchors were developed. They can be obtained from the authors.
