# Supplementary material for: Adherence to Trained Standards After a Faculty Development Workshop on “Teaching With Simulated Patients”
Source: GMS J Med Educ. 2017 Oct 16;34(4):Doc45. doi: 10.3205/zma001122 (PMC5654119; doi:10.3205/zma001122)
Supplement: Table B1: Percentage frequency of observer's ratings of the checklist items (N=60) Table B2: Percentage frequency of observer’s ratings of overall teaching performance (N=60) [file JME-34-45-s-002.pdf]

ATTACHMENT B

Table B 1

Percentage frequency of observer's ratings of the checklist items (N=60)

No.	Category/ Items	Yes (%)	Partly (%)	No (%)	Not appli-cable(%)
1	***Beginning of session*** *The teacher…*				
1.1	*… asks about teamwork in PBL course.*	20	5	71	3
1.2	*… introduces learning objectives.*	48		48	3
1.3	*… introduces schedule for session.*	51	28	17	3
1.4	*… asks about current issues of group members.*	42	15	40	3
2	***Interaction with simulated patient (SP)*** *The teacher…*				
2.1	*…appoints one student to talk to the SP.*	100		0	0
2.2	*…makes sure the group knows the important facts about the SP-case.*	62	17	17	5
2.3	*… discusses learning objectives of the SP-case in group.*	40	32	23	5
2.4	*… activates prior knowledge of students.*	50	18	28	3
2.5	*… distributes specific observation tasks.*	81		19	0
2.6	*A setting for the simulation is prepared.*	97		3	0
2.7	*… talks to SP prior to simulation.*	97		3	0
2.8	*… stays in his/her role as an observer during simulation.*	100	0	0	0
2.9	*SP leaves the room when simulation is finished.*	100		0	0
2.10	*… makes sure nobody gives feedback until SP is back in the room.*	72	17	10	2
2.11	*A setting for the feedback is prepared.*	60	10	30	0
2.12	*… makes sure feedback is given in the recommended order.*	77	15	8	0
2.13	*… makes sure the student summarizes what she/he has learned.*	25	10	64	0
2.14	*SP is involved in evaluation and discussion.*	62	28	10	0
2.15	*A discussion takes place.*	82		17	2
3	***Feedback culture*** *The teacher…*				
3.1	*… starts with a positive comment.*	70	10	12	8
3.2	*… provides „I-messages"*	78	13	2	7
3.3	*… reports specific observations without interpreting.*	75	15	3	7
3.4	*… combines criticism with constructive suggestions for improvement.*	67	22	2	10
3.5	*… only comments on changeable behaviors.*	92		0	9
3.6	*… limits feedback to a few important comments.*	88	3	2	7

No.	Category/ Items	*Yes (%)*	*Partly (%)*	*No (%)*	*Not appli-cable(%)*
3.7	*All the students follow the rules of giving feedback.*	68	30	0	2
4	***Content***<br>*The teacher…*				
4.1	*… orients him-/herself towards the semester's learning objectives.*	98	2	0	0
5	***Facilitation***<br>*The teacher…*				
5.1	*… acts as a facilitator.*	90	8	2	0
5.2	*… summarizes important aspects.*	46	36	19	0
5.3	*… activates students to participate.*	73	20	5	2
5.4	*…visualizes important aspects.*	38	17	43	2
6	***End of session***<br>*The teacher…*				
6.1	*… initiates a final feedback round.*	52	5	40	3
6.2	*… provides an outlook on the next session.*	42	22	25	12
7	***Structure of session***<br>*The teacher…*				
7.1	*… initiates a break.*	82		17	0
7.2	*… adheres to the timeframe.*	63		37	0

Note. All items which were fulfilled by at least 76% of teachers are colored dark green, items fulfilled by 50 to 75% are colored light green, items fulfilled by 26 to 50% are colored yellow and those fulfilled by less than 26% are colored red. This table excludes the four "subitems" as they were not relevant in all observations. Grey boxes indicate, that rating *partly* was not allowed for those items. Percentages do not add up to 100% because of rounding.

Table B 2

Percentage frequency of observer's ratings of overall teaching performance (N=60)

Rating	1	2	3	4	5
%	30	47	20	3	0

Note. Rating from 1 = very good to 5 = very poor.
